# Supplementary material for: Long-Term Clinical Outcome in Systemic Lupus Erythematosus Patients Followed for More Than 20 Years: The Milan Systemic Lupus Erythematosus Consortium (SMiLE) Cohort
Source: J Clin Med. 2022 Jun 22;11(13):3587. doi: 10.3390/jcm11133587 (PMC9267338; doi:10.3390/jcm11133587)

Supplemental Table S1 – SLICC/ACR-DI items at the last time observation

Item	Description	Value (n=221)
1	Cataract	33 (14.9%)
2	Retinal involvement	15 (6.8%)
3	Cognitive impairment/psychosis	12 (5.4%)
4	Seizures	13 (5.9%)
5	Ictus	11 (5%)
6	Ictus+1	3 (1.4%)
7	Cranial /periph neuropathy	11 (5%)
8	Transverse Myelitis	1 (0.5%)
9	GFR<50	20 (9%)
10	ProUr>3.5g/24h	7 (3.2%)
11	ESRD	2 (0.9%)
12	PAH	4 (1.8%)
13	Pulmonary fibrosis	8 (3.6%)
14	Shrinking lung	1 (0.5%)
15	Pleural fibrosis	0 (0%)
16	Pulmonary infarction	0 (0%)
17	Angina-bypass	7 (3.2%)
18	Myocardial infarction + 1	5 (2.3%)
19	Myocardial infarction	11 (5%)
20	Cardiomyopathy	8 (3.6%)
21	Valvular disease	12 (5.4%)
22	Chronic pericarditis	2 (0.9%)
23	Claudicatio	1 (0.5%)
24	Minor tissue loss	6 (2.7%)
25	Tissue loss	1 (0.5%)
26	Tissue lost+1	1 (0.5%)
27	DVT	12 (5.4%)
28	lower GI tract, liver, spleen surgery / infarction	3 (1.4%)
29	lower GI tract, liver, spleen surgery / infarction +1	0 (0%)
30	Mesenteric insufficiency	0 (0%)
31	Chronic peritonitis	2 (0.9%)
32	Stricture / upper GI tract surgery	1 (0.5%)

33	Myopathy/atrophy	5 (2.3%)
34	Erosive/deforming arthritis	26 (11.8%)
35	osteoporosis fracture	25 (11.3%)
36	Avascular necrosis	6 (2.7%)
37	Avascular necrosis+1	3 (1.4%)
38	Osteomyelitis	1 (0.5%)
39	Alopecia	5 (2.3%)
40	Scarring/panniculum	10 (4.5%)
41	Skin ulcers	4 (1.8%)
42	Gonadal failure	15 (6.8%)
43	Diabetes	8 (3.6%)
44	Malignancy	20 (9%)
45	Malignancy+1	4 (1.8%)

Supplemental Figure S1 – BILAG scores at relevant time-points

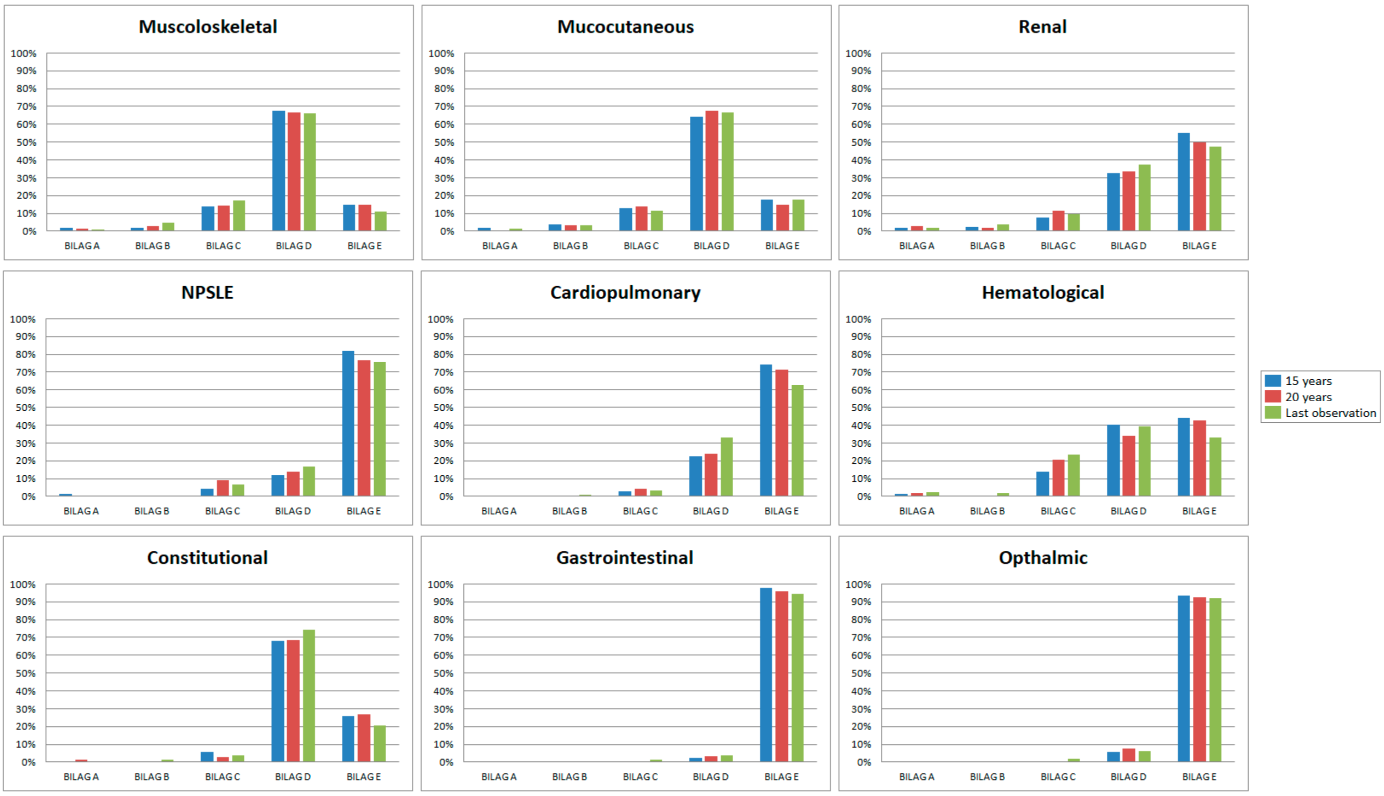

Percentage of patients with BILAG A-to-E scores in each domain at the 15th, 20th year of disease or at the last observation. Data from 168 patients at 15 years, 146 patients at 20 years; full dataset was available at the last observation.

Supplemental Figure S2 – Risk of flares according to use of steroids at the 20th year or thereafter

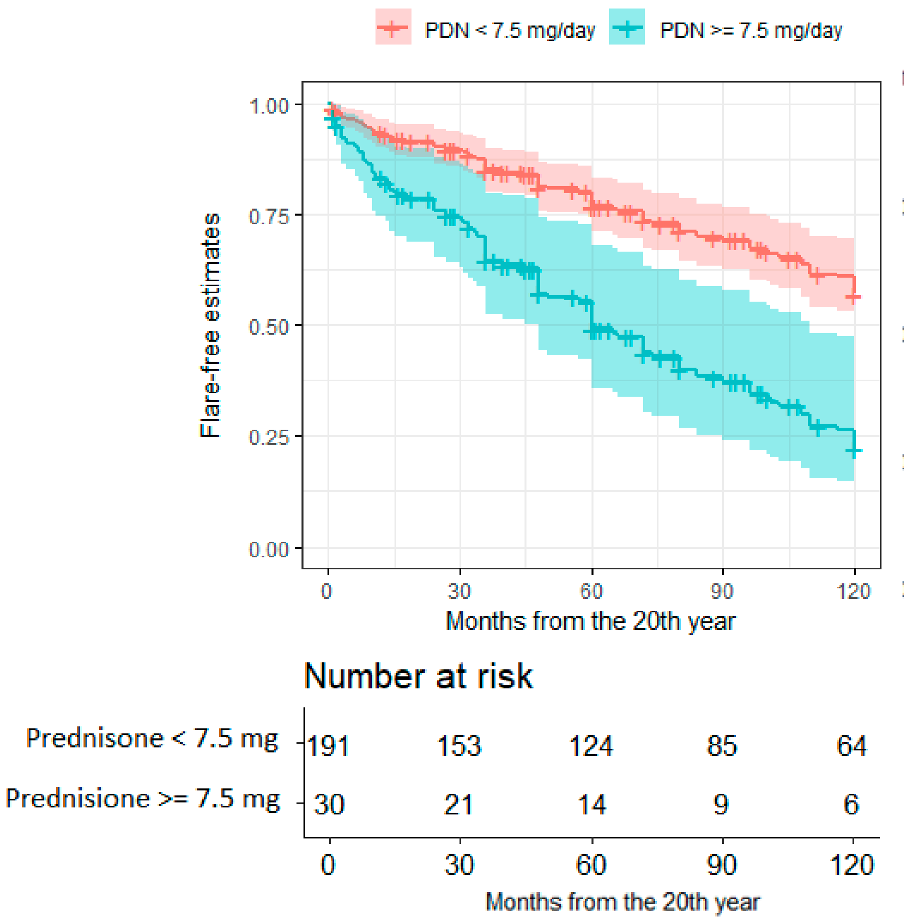

Supplement: Supplementary file 1 [file jcm-11-03587-s001.zip › jcm-1720863-supplementary.pdf]
